# Post-transplant recurrence of steroid resistant nephrotic syndrome in children: the Italian experience

**DOI:** 10.1007/s40620-019-00660-9

**Published:** 2019-10-15

**Authors:** William Morello, Sairaj Puvinathan, Giuseppe Puccio, Gian Marco Ghiggeri, Luca Dello Strologo, Licia Peruzzi, Luisa Murer, Michela Cioni, Isabella Guzzo, Enrico Cocchi, Elisa Benetti, Sara Testa, Luciana Ghio, Gianluca Caridi, Massimo Cardillo, Rosanna Torelli, Giovanni Montini

**Affiliations:** 1 Pediatric Nephrology, Dialysis and Transplant Unit, Department of Clinical Sciences and Community Health, Fondazione IRCCS Ca’ Granda-Ospedale Maggiore Policlinico, University of Milan, via della Commenda, 9, 20122 Milan, Italy; 2 Division of Nephrology, Dialysis, and Transplantation, Scientific Institute for Research and Health Care, IRCCS Istituto Giannina Gaslini, Genoa, Italy; 3 Renal Transplant Unit, Bambino Gesù Children’s Research Hospital IRCCS, Rome, Italy; 4 Pediatric Nephrology Unit, Regina Margherita Children’s Hospital, Città della Salute e della Scienza di Torino, Turin, Italy; 5 Pediatric Nephrology, Dialysis and Transplant Unit, Department of Women’s and Children’s Health, Hospital-University of Padova, Padua, Italy; 6 North Italy Transplant program (NITp), UOC Coordinamento Trapianti, Fondazione IRCCS Ca’ Granda-Ospedale Maggiore Policlinico, Milan, Italy

**Keywords:** Steroid-resistant nephrotic syndrome, Kidney transplant, Post-transplant recurrence

## Abstract

**Background:**

Steroid resistant nephrotic syndrome (SRNS) is a frequent cause of end stage renal disease in children and post-transplant disease recurrence is a major cause of graft loss.

**Methods:**

We identified all children with SRNS who underwent renal transplantation in Italy, between 2005 and 2017. Data were retrospectively collected for the presence of a causative gene mutation, sex, histology, duration of pre-transplant dialysis, age at onset and transplant, HLA matching, recurrence, therapy for recurrence, and graft survival.

**Results:**

101 patients underwent a first and 22 a second renal transplant. After a median follow-up of 58.5 months, the disease recurred on the first renal transplant in 53.3% of patients with a non-genetic and none with a genetic SRNS. Age at transplant > 9 years and the presence of at least one HLA-AB match were independent risk factors for recurrence. Duration of dialysis was longer in children with relapse, but did not reach statistical significance. Overall, 24% of patients lost the first graft, with recurrence representing the commonest cause. Among 22 patients who underwent a second transplant, 5 suffered of SRNS recurrence. SRNS relapsed in 5/9 (55%) patients with disease recurrence in their first transplant and 2 of them lost the second graft.

**Conclusions:**

Absence of a causative mutation represents the major risk factor for post-transplant recurrence in children with SRNS, while transplant can be curative in genetic SRNS. A prolonged time spent on dialysis before transplantation has no protective effect on the risk of relapse and should not be encouraged. Retransplantation represents a second chance after graft loss for recurrence.

## Introduction

Steroid resistant nephrotic syndrome (SRNS) is the most common acquired cause of end stage renal failure (ESRD) requiring transplantation in children. Advances in genetic screening have allowed the identification of a monogenic cause of SRNS in one-third of cases [1]. Genetic SRNS are associated with an underlying mutations in genes encoding podocyte associated proteins, resulting in structural or functional disruption of the glomerular filtration barrier [2]. The pathophysiology of SRNS without underlying mutations remains poorly explained and is thought to involve an unknown circulating permeability factor [3] which may also be implicated in the recurrence soon after transplantation [4].

Unfortunately, in up to 50% of patients, SRNS relapses after transplantation and disease recurrence is a major cause of graft loss [4–6]. Genetic SRNS have been reported to have a low rate of recurrences [7–9]. On the other hand, previous studies have suggested non-African race, rapid progression to ESRD (< 3 years) and previous recurrence after transplantation to be associated with SRNS relapse [8, 10, 11], but no established risk factors can actually predict the outcome. A longer time on dialysis before transplantation was believed to decrease the risk of relapse, but not confirmed by large reports [12, 13].

There is currently little consensus regarding the best management of post-transplant SRNS recurrence, which represents a devastating complication for families and physicians, and poses a significant threat to allograft survival. Plasma exchange (PE), steroids and rituximab are the most common strategies to treat the recurrence [14].

The objective of this study was to identify factors affecting the risk of recurrence and graft loss in children with SRNS, by stratifying the population according to their genetic status.

## Methods

We performed a retrospective, multicentre, observational cohort study to address the long-term prognosis of renal graft, the risk factors for recurrence and the predictors for response to therapy following recurrence in paediatric patients undergoing renal transplantation because of a SRNS.

We identified patients who underwent renal transplantation at all five Italian paediatric transplant centres, between 2005 and 2017, with a primary diagnosis of SRNS and onset before 18 years. Patients were included if a clinical diagnosis of SRNS was made in an individual with otherwise unexplained nephrotic-range proteinuria refractory to standard steroid therapy and subsequently confirmed by renal biopsy showing a histological picture of focal segmental glomerulosclerosis (FSGS), minimal change disease (MCD), or diffuse mesangial sclerosis (DMS). Clinical records, pathology reports and genetic screening results were reviewed for the purposes of this study. Data were also collected about sex, age of disease onset, duration of pre-transplant dialysis, age at transplant, immunosuppression, allograft donor characteristics, disease recurrence, therapy for recurrence, and graft survival.

Patients were divided in three groups: Group A (Genetic SRNS): patients with an identified causative genetic mutation and/or a first degree relative with SRNS and/or extra-renal disease manifestations pathognomonic of SRNS, Group B (Idiopathic SRNS): patients with a negative or heterozygous recessive genetic test result and without a first degree family history or associated extra-renal manifestations pathognomonic of SRNS, Group C (unknown genetic status): patients with no genetic analysis performed and no family history or extra-renal manifestations typical of SRNS.

### Definitions

Nephrotic-range proteinuria, urine protein:creatinine ratio (uPr/uCr) ≥ 2 mg/mg. Age at disease onset, age at first clinical presentation of nephrotic syndrome. Steroid resistance, persistence of nephrotic range proteinuria following 4 weeks of daily 60 mg/sqm prednisone therapy. Post-transplant disease recurrence, an otherwise unexplained persistent nephrotic range proteinuria after renal transplantation, when rejection was excluded. Graft loss, functional failure of the renal allograft, necessitating renal replacement therapy. Remission after recurrence, complete resolution of proteinuria (uPr/uCr < 0.2 mg/mg). Partial remission after recurrence, persistent reduction of proteinuria (uPr/uCr < 2 mg/mg) with preserved renal function.

### Statistical analysis

Categorical variables were compared using the Chi squared test for independence. The distribution of continuous variables in groups was compared using the Wilcoxon signed-rank test and the Kruskal–Wallis test. Linear regression models were used to compare continuous variables. For multivariate analysis, multiple logistic regression models were used. A *p* value < 0.05 was considered statistically significant. All statistical analyses were performed using the open source software R. (R Core Team, 2014. R: A language and environment for statistical computing, R Foundation for Statistical Computing, Vienna, Austria).

## Results

### Study cohort

**Fig. 1:**
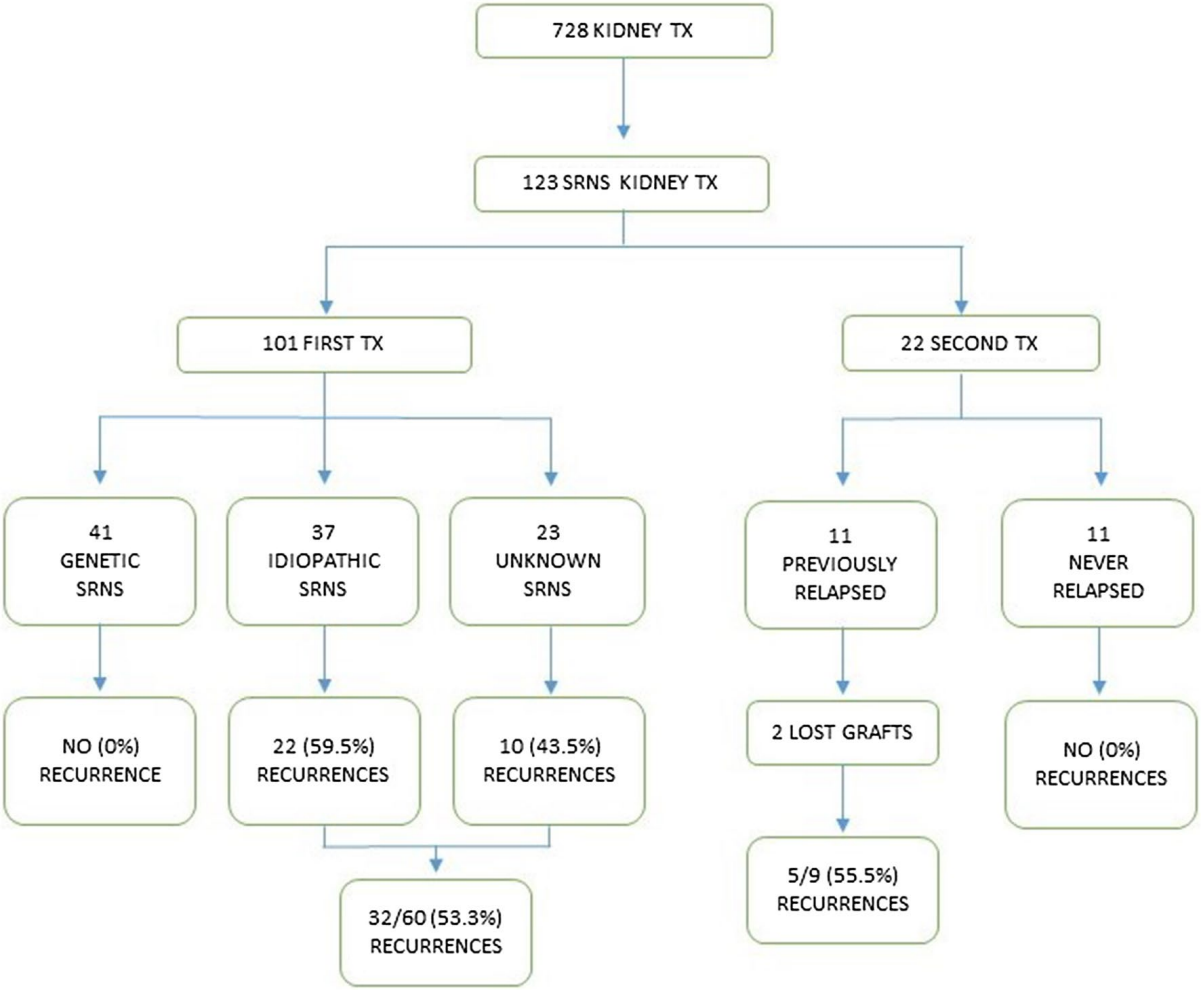
Study cohort

During the study period, a total of 728 (618 deceased and 110 living donors) renal grafts were performed at the 5 Italians pediatric transplant centres, of whom 123 in patients with ESRD secondary to SRNS. 101 patients received a first renal allograft and 22 a second renal transplant (12 failures of the original cohort and 10 failures of a first transplant that occurred before the study period). The number of patients who received a first transplant at each center is as follows: Istituto G. Gaslini, Genova—31, Bambino Gesù Children’s Hospital, IRCCS, Rome—23, Fondazione IRCCS Ca’ Granda, Ospedale Maggiore, Milan—20, Regina Margherita Children’s Hospital, Turin—15, University Hospital of Padua—12. The study cohort is summarized in Fig. 1.

### First renal graft

101 patients (52.5% males) underwent a first renal transplant. The median age at onset was 2.8 years of age (range 0–17.2); 24 individuals (25.2%) presented with congenital SRNS, defined as onset of disease within the first 3 months of life. Renal histology was consistent with FSGS in 85 cases, MCD in 14 and DMS in 2. Main demographic and clinical characteristics are summarized in Table 1. At transplant all patients received an induction therapy with basiliximab and immunosuppression with steroids, calcineurin inhibitors and mofetil mycophenolate. Two patients were treated with plasmapheresis pre-transplantation.

**Table 1:** Main demographic and clinical characteristics of SRNS children transplanted between 2005 and 2017

Characteristics	First renal Tx	Second renal Tx
Total	101	22
Gender		
Male	53 (52.5%)	13 (59.1%)
Female	48 (47.5%)	9 (40.9%)
Genetic disease		
Yes	41 (40.6%)	5 (22.7%)
No	37 (36.6)	13 (56.6%)
Unknown	23 (22.8%)	4 (18.2%)
Age at onset (years) median (range)	2.8 (0–17.2)	4.45 (0–14.29)
Age at transplant (years) median (range)	11.8 (2.6–20.8)	16.71 (4.56–31.1)
Time to ESRD (years) median (range)	3.3 (1.7–14.3)	2.5 (0–7.5)
Time on dialysis before transplantation (years) median (range)	2 (0–9)	Not available
Donor type		
Living	6 (5.9%)	2 (9.1%)
Deceased	95 (94.1%)	20 (90.9%)
Follow-up (months) median (range)	58.5 (0.7–157.8)	40 (0–148)

Genetic testing results were available for 76 individuals (75.2%) (Table 2): 39 had an autosomal dominant mutation or were homozygous for a recessive mutation, 8 were heterozygous carriers and 29 had a negative genetic test. Genetic results were unavailable for 25 patients, among whom we were able to identify 2 additional patients with genetic SRNS: one had a sibling with established genetic SRNS and another showed extra-renal disease manifestations suggesting a genetic disease. The patient cohort was therefore comprised of: Group A (genetic SRNS): 41 individuals (40.6%), Group B (idiopathic SRNS): 37 individuals (36.6%), and Group C (unknown genetic status): 23 individuals (22.8%). Age at the onset was similar between Group B (idiopathic) and Group C (unknown), while it was younger for patients with a genetic disease (Group A) (p < 0.0001).

**Table 2:** Prevalence of mutations among patients with available genetic results

Gene	Encoded protein	Mode of inheritance	Genetic tests n = 76
NPHS1	Nephrin	Recessive	13
WT1	Wilms tumour protein	Dominant	11
NPHS2	Podocin	Recessive	8
ACTN4	α-Actinin	Dominant	2
PLCE1	Phospholipase C	Recessive	1
COL4A5	Type IV collagen α5 chain	X-linked recessive	1
SMARCAL1	SWI/SNF-related, matrix-associated, actin-dependent regulator of chromatin, subfamily A-like protein 1	Recessive	1
LMX1B	LIM homeobox transcription factor 1 β	Dominant	1
COQ2	Coenzyme Q2	Recessive	1
Heterozygous carriers		Recessive	8
No mutations			29

NPHS1, encoding nephrin, was the single most commonly mutated gene and accounted for one-third (33.3%) of positive genetic results, followed by WT1, encoding Wilms tumour protein and NPHS2, encoding podocin. Mutations in these genes were responsible of 28.2% and 20.5% of genetic SRNS, respectively. Taken together, mutations in NPHS1, NPHS2, and WT1 accounted for 82% of identified genetic cases. Pathogenetic mutations were also identified in the following genes: PLCE1, ACTN4, COL4A5, SMARCAL1, LMX1B, COQ2 (Table 2). In eight cases renal disease was associated with a syndromic presentation, as follows: Denys Drash syndrome in four cases and one case each of Frasier syndrome, WAGR syndrome, Leopard syndrome and Schimke immuno-osseous dysplasia.

### Post-transplant disease recurrence

Median follow-up is 58.5 months (range 0.7–157.8). SRNS recurred in 32 individuals (31.7%) after the first renal transplant, at a median time of 2 days post-transplantation. When stratified by genetic status, the incidence of post-transplant disease recurrence was 59.5% in Group B (idiopathic SRNS) and 43.5% in Group C (unknown genetic status). No Group A (genetic SRNS) child experienced disease recurrence and this group was therefore excluded from further analysis (Table 3).

**Table 3:** Incidence of recurrence, stratified by genetic testing results

Characteristics	Total, n = 101	Recurrence, n = 32	No recurrence, n = 69
Genetic results *n,* %			
Negative	37	22 (59.5)	15 (40.5)
Unknown	23	10 (43.5)	13 (57.5)
Positive	41	0 (0.0)	41 (100.0)

The difference in post-transplant disease recurrence between Group B and C, however, was not significant (p = 0.23). Risk factors for recurrence were evaluated in the remaining 60 patients (Group B and C). Overall, SRNS recurred in 32/60 (53.3%) non-genetic patients.

**Fig. 2:**
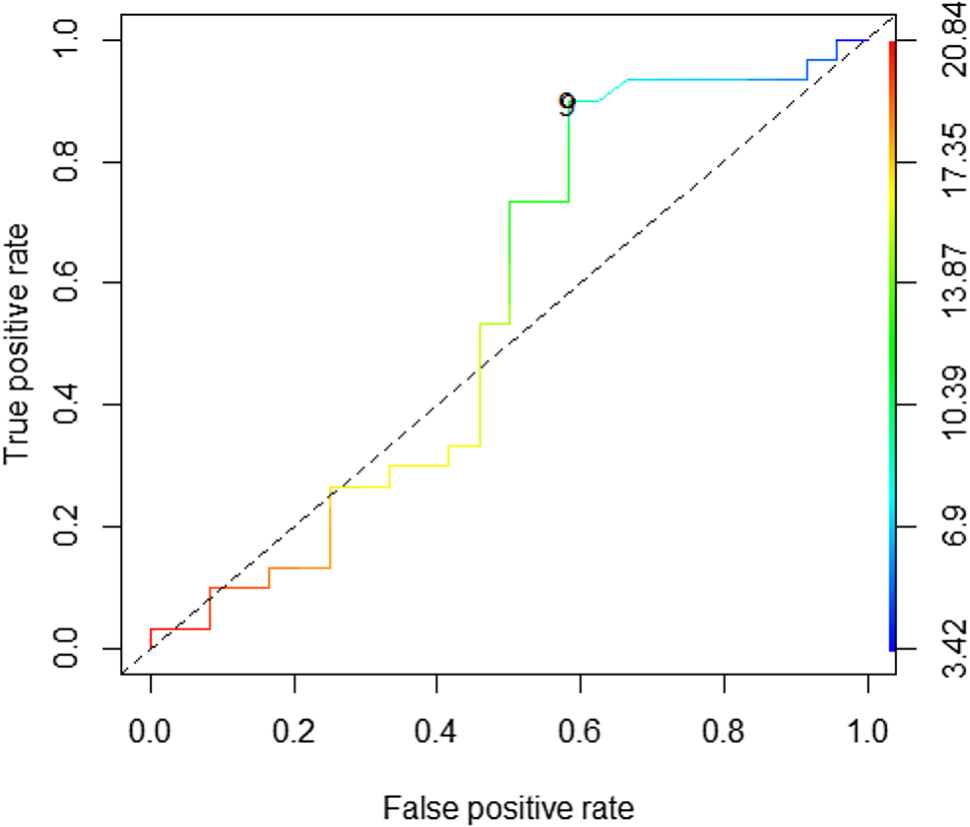
ROC curve identifying the best cut-off for age at transplant with FPR = 0.58333333, TPR (sensitivity) = 0.86666667, Specificity = 0.41666667, p value = 0.01823

As all relapses except one (identified 10 years after transplantation) occurred within 8 months from transplant, the analysis was made at 8 months of follow-up and included all evaluable patients (54 patients). Age at transplant was categorized as ≥ 9 years, following a ROC analysis identifying it as the best cut-off for relapse prediction in our dataset (Fig. 2). Bivariate analysis was performed by Wilcoxon test for independent samples. Multivariate analysis was performed by a logistic regression model.

At bivariate analysis, the following variable were significantly associated to relapse:

- Age ≥ 9 years (p = 0.01823)
- At least one HLA AB match (p = 0.01752)
- At least one HLA DR match (p = 0.01763)

Gender, donor age and donor type (living or deceased) did not affect the risk of recurrence. Time to ESRD and duration of dialysis before transplant were not significantly associated with relapse; anyway, they were both longer in children with relapse (median = 4.6 vs 2.7 years, p = 0.2673 and 2.4 vs 1.8 years, p = 0.06582, respectively).

We were not able to assess the role of different induction schedules, since all patients were homogeneously treated with basiliximab and immunosuppressive therapy. However, among the two patients treated with pre-transplantation PE, one experienced relapse the day after transplant.

Multivariate analysis included all the variables associated with recurrence at the bivariate analysis with a p value < 0.1 (Table 4). Age at transplant > 9 years and HLA-AB match were the only independent risk factors for recurrence after transplant (p = 0.01017 and p = 0.02465, respectively). However, the best prediction model for relapse, characterized by the lowest residual deviance and lowest AIC, included also a longer duration of dialysis before transplant (Null deviance: 68.029 on 49 degrees of freedom; Residual deviance: 49.584 on 46 degrees of freedom; AIC: 57.584; p = 0.06994). The model including the aforementioned 3 variables has a likelihood ratio test p value of 0.000356, and a pseudo R squared value of 0.271136 (McFadden method).

**Table 4:** Variables included in the multivariate analysis

Variables
Age ≥ 9 years
At least one HLA AB match
At least one HLA DR match
Duration of dialysis before transplant

The risk factors for recurrence in Group B and C are summarized in Table 5.

**Table 5:** Risk factors for post-transplant disease recurrence in Group B (idiopathic SRNS) and Group C (unknown genetic status) individuals, at 8 months of follow-up

Variables	Total	Recurrence, n (%)	No recurrence, n (%)	Univariate analysis *p* value	Multivariate analysis *p* value
Gender					
Male	30 (55.6%)	18 (60%)	12 (40%)	0.4624	0.26990
Female	24 (44.4%)	12 (50%)	12 (50%)		
Age at transplant, *yr*					
≥ 9	40 (74.0%)	26 (65%)	14 (35%)	**0.01823**	**0.01017**
< 9	14 (26.0%)	4 (28.6%)	10 (71.4%)		
HLA-AB matching				0 vs > 0	0 vs > 0
0	7 (13.5%)	1 (14.3%)	6 (65.7%)	**0.01752**	**0.02465**
1	18 (34.6%)	13 (72.2%)	5 (27.8%)		
2	17 (32.7%)	9 (52.9%)	8 (47.1%)		
3	10 (19.2%)	6 (60%)	4 (40%)		
HLA-DR matching					
0	16 (30.8%)	5 (31.2%)	11 (68.8%)	**0.01763**	0.46309
1	34 (65.4%)	24 (70.6%)	10 (29.4%)		
2	2 (3.8%)	0 (0%)	2 (100%)		

	Recurrence	No recurrence		
Duration of dialysis median, range	2.4, 0.6–9	1.8, 0.1–5	0.06582	0.06994
Time to ESRD median, range	4.6, 0–12	2.7, 0–13	0.2673	0.72323
Donor age median, range	14, 1–63	11, 1–56	0.1609	0.84874

Statistically significant values in bold

Disease recurrence was treated in all patients with PE with a median of 20 sessions (range 4–79). 22 were treated with rituximab and 9 with high dose steroids. The use of other therapeutic agents was as follows: ofatumumab (3), mesenchymal stromal cells (2), intravenous immunoglobulins (2), abatacept (2), cyclophosphamide (2), cyclosporin (1) and thymoglobulin (1). Overall, a complete or partial remission was achieved in 15 and 4 patients, respectively, 13 patients (40.6%) failed to achieve sustained disease remission, despite treatment and 11/13 subsequently lost the graft. Use of rituximab or high dose steroids did not influence the response rate (p = 0.3574). The remission was persistent with preserved renal function in 13/15 patients. One patient had second untreatable relapse 10 years after transplant and lost the kidney. One additional patient with partial remission, following experienced rejection and lost his graft.

### Graft loss

24 patients (23.8%) experienced loss of a first renal graft. The causes of graft loss were as follows: disease recurrence in 12 (50%), rejection in 6 (25%), primary non-functioning graft in 3 (12.5%), thrombosis in 2 (8.3%) and chemotherapy toxicity for post-transplant thrombo-proliferative disease in one case (4.1%). In addition, death with a functional graft due to sepsis occurred in two patients.

### Second renal transplant

During the study period, 22 SRNS patients received a second renal graft; 11 of them had had a recurrence in the first graft, while 11 lost their transplant for different reasons. Among patients with a previous recurrence, 2/11 patients lost their graft immediately after the transplantation for reasons different from relapse (death and surgery complications) and were not included in the following analysis. After a median follow-up of 40 months, five patients relapsed on the second transplant. All of them have had a recurrence in the first graft. Therefore, in our population, only 5 out 9 (55.5%) evaluable patients with a previous relapse experienced recurrence of proteinuria after the second kidney transplantation. Among five relapsed patients, only two subsequently lost the second graft. None of the 11 patients who lost the first transplant for different reasons suffered of relapse, with 4/11 having a genetic disease. Outside SRNS recurrence, two patients experienced a graft rejection and lost the second kidney graft and one patient died after a post-transplant lymphoproliferative disease.

## Discussion

SRNS is a leading cause of ESRD in children. Post-transplant recurrence is a common complication, associated with an increased risk of graft loss. Many efforts have been made to identify the risk factors for recurrence in order to improve prevention and treatment strategies [5, 6, 15].

Our study gives a clear picture of the Italian experience with kidney transplantation in children with SRNS, during a period of over 10 years, encompassing all the recent acquisition regarding the etiopathology and therapeutic options for SRNS.

The overall incidence of post-transplant disease recurrence (53% of non-genetic patients) is consistent with the available scientific literature, stretching back almost three decades [16, 17]. Recurrence is confirmed to be a very early event, with a median time from transplant of 2 days and 30/32 events occurring within the first 2 months after transplantation. By stratifying the cohort according to genetic status, we have been able to confirm that genetic SRNS does not recur after transplant. While previous reports have identified the genetic status as an important risk factor for disease recurrence, most available retrospective studies are unable to account for the genetic status of the majority of their cohorts [17, 18]. To the best of our knowledge, no previous studies were able to assess the risk of recurrence in an equally characterized population, as regards genetic disease. In our study, genetic results were indeed available for 76/101 patients (75.2%), furthermore since the remaining individuals (Group C, unknown genetic status) closely resemble Group B (Idiopathic SRNS) in key clinical features, including similar age of onset and rate of recurrence (43.5%), it is likely that most of them also represent cases of idiopathic SRNS. Indeed, we believe that patients with early onset or congenital SRNS were more likely to be tested for a genetic disease, while genetic testing was less performed in older children and adolescents with a clinical picture of idiopathic SRNS.

Our observation is in line with previous studies that report no or very low relapse rate after transplantation in children with genetic SRNS [9, 17, 19]. Few old reports have suggested a risk of relapse for genetic SRNS, but they are almost all related to NPHS2 mutation, including heterozygous individuals [20–23]. The causative role of the variants included in these reports should be reconsidered, as exquisitely suggested in a recent review by Bierzynska [15].

The rate of recurrence in idiopathic SRNS (Group B) was 59.5%. The result is slightly superior than previously reported. When genetic patients are excluded, Ding [17] and Pelletier [19] found a relapse rate of 46.3% and 47%, respectively. The lower recurrence rate described by these groups could be justified by the presence of unknown genetic SRNS patients. Indeed, when both Group B and C are considered, the overall rate of recurrence was 53.3% in our cohort. Therefore, our data underline the importance of a genetic evaluation for SRNS genes in order to plan transplantation, as it represents the principle risk factor for recurrence.

Aside from absence of a genetic aetiology, our study identified age at transplant greater than 9 years and HLA-AB match as independent risk factors for recurrence. The best prediction model for recurrence included also a longer duration of dialysis.

Nehus et al. reported a higher rate of recurrence in younger children, among a cohort of 327 patients, though genetic results were not reported for any participants [24]. No significant difference in relapse rate according to the age at transplant were detected by Tejani and Stablein [12] and in the more recent studies by Ding et al. and Pelletier et al. [17, 19]. Again, unavailability of genetic testing for the majority of their patients could justify the different findings.

HLA-AB match was independently related to recurrence in our cohort, in contrast HLA AB or DR match did not influence the risk of relapse in the study by Tejani et al. [12] and did not affect transplant outcome in adolescent with SRNS in a retrospective study of the NAPRTCS registry [25].

Following the evidence that a circulating factor is responsible for recurrence, it has been suggested that a prolonged dialysis prior to renal transplantation would have a protective effect as far as the risk of relapse is concerned. The results of our study do not support this hypothesis. Indeed, in our study cohort, a longer duration of dialysis was associated with an increased risk of relapse. Even if this variable did not reach significance, its inclusion identifies the best prediction model for recurrence (R^2^ 0.271136). Among the few studies which investigated the association between duration of dialysis and disease recurrence, no significant differences were found in a single centre experience of 43 patients by Senggutuvan [16]. In a larger cohort of 132 paediatric renal transplants, found no relationship between disease recurrence and duration of dialysis was found [12]. Hence, since no protective effect was proven by others and our data show a longer duration of dialysis in patients with recurrence, even if not statistically significant, it is not justified to prolong the duration of dialysis before transplantation in children with SRNS.

Whether donor type (living vs deceased) is significantly associated with disease recurrence remains controversial. Data from old registries [26, 27] found no increased recurrence rates according to the type of donors. Other studies have suggested living donor as an independent risk factor for recurrence [17, 28, 29]. Our study cohort included only six living donor recipients (5.9%), reflecting the reluctance of paediatric nephrologists to use living donors in SRNS patients, due to the risk of recurrence and graft loss. On the other hand, since in our cohort no relapses occurred in genetic SRNS, another important clinical implication of our study is that living kidney donors can be safely used in genetic SRNS patients.

All patients from our cohort were treated with PE, following SRNS recurrence. Complete or partial remission was achieved in 19/32 (59.4%) children, with a functioning graft after a median follow-up of 39.5 months. Similar rates of response were previously reported. Kashgary et al. in their meta-analysis identified a remission rate of 70.2% in children treated with PE [14]. A lower response rate was reported by Pelletier [19], but remission information was available only for 49/64 (77%) relapsed patients and the detailed immunosuppressive strategy is missing. According to our results, PE is confirmed as an effective treatment for recurrence. Even the small numbers, rituximab and high dose steroids did not influence the response rate in our cohort.

On the other hand, disease recurrence was the leading cause of graft loss in non-genetic SRNS and the rate of graft loss after relapse (34.3%) in our study is consistent with previous data [26].

Among the small number of retransplanted individuals included in our study, the overall incidence of relapse in a second renal graft after a first recurrence is not significantly different from the first transplant (55%). In 4/9 patients who experienced a relapse in their first transplant, proteinuria did not recur after the second transplantation. This contrasts with reported small cohorts in whom the incidence of recurrence approaches 100% once the first transplant was lost for recurrent SRNS [10, 12, 28, 30]. We are not able to identify the factors responsible of the different outcome, but according to our data retransplantation after relapse can be considered in children with SRNS.

## Conclusions

Twelve years of the Italian experience with post-transplant SRNS recurrence allows us to reach different important conclusions. Firstly, the absence of underlying genetic mutations predicts a high risk of post-transplant recurrence, therefore genetic screening must be performed in all children with SRNS before transplantation in order to best plan their care in the post-transplant period. Age > 9 years is an independent risk factor for recurrence, while a prolonged time spent on dialysis before transplantation has no protective effect on the risk of relapse and should not be encouraged. Living donor did not influence the risk of relapse and can be safely used in genetic SRNS patients. PE based treatment strategies are effective in the majority of relapsed patients. Finally, in those who experience graft loss, even for recurrence, it is appropriate to consider retransplantation, as it maybe curative in the long term.
